# Exosomal lncRNA HOTAIR induce macrophages to M2 polarization via PI3K/ p-AKT /AKT pathway and promote EMT and metastasis in laryngeal squamous cell carcinoma

**DOI:** 10.1186/s12885-022-10210-5

**Published:** 2022-11-24

**Authors:** Jingting Wang, Nan Wang, Zeyu Zheng, Yanlu Che, Masanobu Suzuki, Satoshi Kano, Jianguang Lu, Peng Wang, Yanan Sun, Akihiro Homma

**Affiliations:** 1 Department of Otorhinolaryngology, Head and Neck Surgery, The Second Affiliated Hospital, Harbin Medical University, Harbin, China; 2 Department of Otolaryngology-Head and Neck Surgery, Hokkaido University Graduate School of Medicine, Sapporo, Japan

**Keywords:** lncRNA HOTAIR, Exosomes, Macrophage, LSCC, EMT

## Abstract

Exosomes are a new way of the communication between the tumor cell and macrophage in the micro-environment. The macrophage can be induced to different phenotypes according to the different tumors. In the present study, long-chain noncoding RNA HOTAIR (lncRNA HOTAIR) was highly expressed in LSCC and exosomes. The pathway of exosomal lncRNA HOTAIR inducing macrophage to M2 polarization in the LSCC was investigated. The carcinoma tissues and adjacent tissues were collected from 104 LSCC cases, and the positive relationship between CD163-/CD206-M2 macrophage infiltration and clinical phase, lymph node spreading and pathological phase in LSCC was observed. To examine the role of exosomal lncRNA HOTAIR, macrophages were co-cultured with LSCC-exosomes of high lncRNA HOTAIR expression or transferred with HOTAIR mimics. It was suggested that exosomal lncRNA HOTAIR can induce macrophages to M2 polarization by PI3K/p-AKT/AKT signaling pathway. Furthermore, exo-treated M2 macrophages facilitate the migration, proliferation, and EMT of LSCC.

**Supplementary Information:**

The online version contains supplementary material available at 10.1186/s12885-022-10210-5.

## Introduction

Laryngeal squamous cell carcinoma(LSCC) is a highly invasive malignant tumor of head and neck. In 2012, there were 157,000 new cases in the world, accounting for 1.1% of new carcinoma cases, and 83,000 total deaths worldwide [1]. The latest statistics of China show 26,400 newly diagnosed cases and 14,500 dead cases in 2015. Despite the remarkable achievements in diagnosing and treating processes, the surviving ratio remains not optimistic, and the 5-year surviving ratio has fallen (66%-63%) over the last four decades [2]. Recurrence and metastasis as major factors in tumors limit their successful treatment. Approximately 60% of cases in laryngeal carcinoma are already advanced in carcinoma for the first visit (stage III or IV) [3]. Therefore, the need for further research and innovation in the field is meaningful.

As the tumor grows, the malignant tumor cells interact with adjacent parenchymal cells, stromal cells and immune cells, and combine with soluble mediators and extracellular matrix to form tumor micro-environment (TME) [4]. The interaction between immune cells and tumor cells runs through the whole process of tumor initiation, growth and diffusion [5]. Nevertheless, macrophages are often the main immune cells within tumor micro-environment [6]. In tumor micro-environment, according to the various tumor progression stages and different stimulating factors, macrophage can differentiate into M1 and M2 phenotypes [7–9]. M1 macrophages primarily play an anti-tumor role to promote inflammatory response and synthesize some killing cytokines (ROI, TNF, Nitric Oxide (NO), e.g.). M2 macrophages can be reduced to different types under different stimuli, and can inhibit inflammatory response and produce factors such as IL-10 and TGF-β. Moreover, it promotes the angiogenesis of inflammatory response, as well as tumor growth and metastasis. However, in many human cancers, elevated M2 macrophage levels are associated with increased morbidity and poor survival, demonstrating that the differentiation hypotypes of macrophages can effectively guide the clinical treatment of tumors [10].

Exosomes are circular fragments of membrane released from the endosomal compartment, secreted by various cells including carcinoma cells, which are considered to be critical to transmit genetic information between tumor cells and interstitial cells [11]. As reported in recent studies, exosomes, as a new way of communication between tumor cells and macrophages, mediate different hypotypes of macrophage with various sources of tumor cells [12]. As suggested by Ying Xiang et al., epithelial ovarian carcinoma can differentiate macrophages into M2 via the exosomes pathway, which can promote the proliferation and migration of tumor cells. In existing studies, a high expression of lncRNA HOTAIR was found in tissues and cells in LSCC. High expression of lncRNA HOTAIR can inhibit PTEN expression through PTEN methylation. The transfection of LSCC cells with si-lncRNA HOTAIR lentivirus lead to inhibited tumor cells proliferation, arrested cells growth, and increased apoptotic cells, and nude mouse tumorigenesis can be significantly inhibited [13]. High expression of lncRNA HOTAIR in exosomes was also found in serum and cell culture medium of LSCC patients. Exosomal lncRNA HOTAIR expression was positively related to TNM stage and clinical stage. Exosomal lncRNA HOTAIR can be a biomarker for LSCC [14]. Xuetao Cao's research group considered that Mir-3473b targeting PTEN protein can reduce the ability of IFN to induce macrophage polarization [15], which demonstrated that IL-10 regulation inhibits the expression of PTEN and induces the polarization of macrophages to M2, thus activating the PI3K/AKT signaling pathway. In GSK3 small glioma, Scriptaid, an HDAC inhibitor, inhibits PTEN phosphorylation by activating PI3K/AKT signaling pathway and induces macrophage polarization to M2 [16].

Therefore, this study will verify that lncRNA HOTAIR of LSCC is delivered to macrophages in the micro-environment by exosomes, thus inducing the polarization of macrophages to M2 by activating the PI3K/p-AKT/AKT signaling pathway. Furthermore, the polarized M2 macrophages can promote the proliferation, migration and EMT in LSCC.

## Results

### Relationship between M2 macrophage expression and clinicopathological parameters in LSCC

**Fig. 1:**
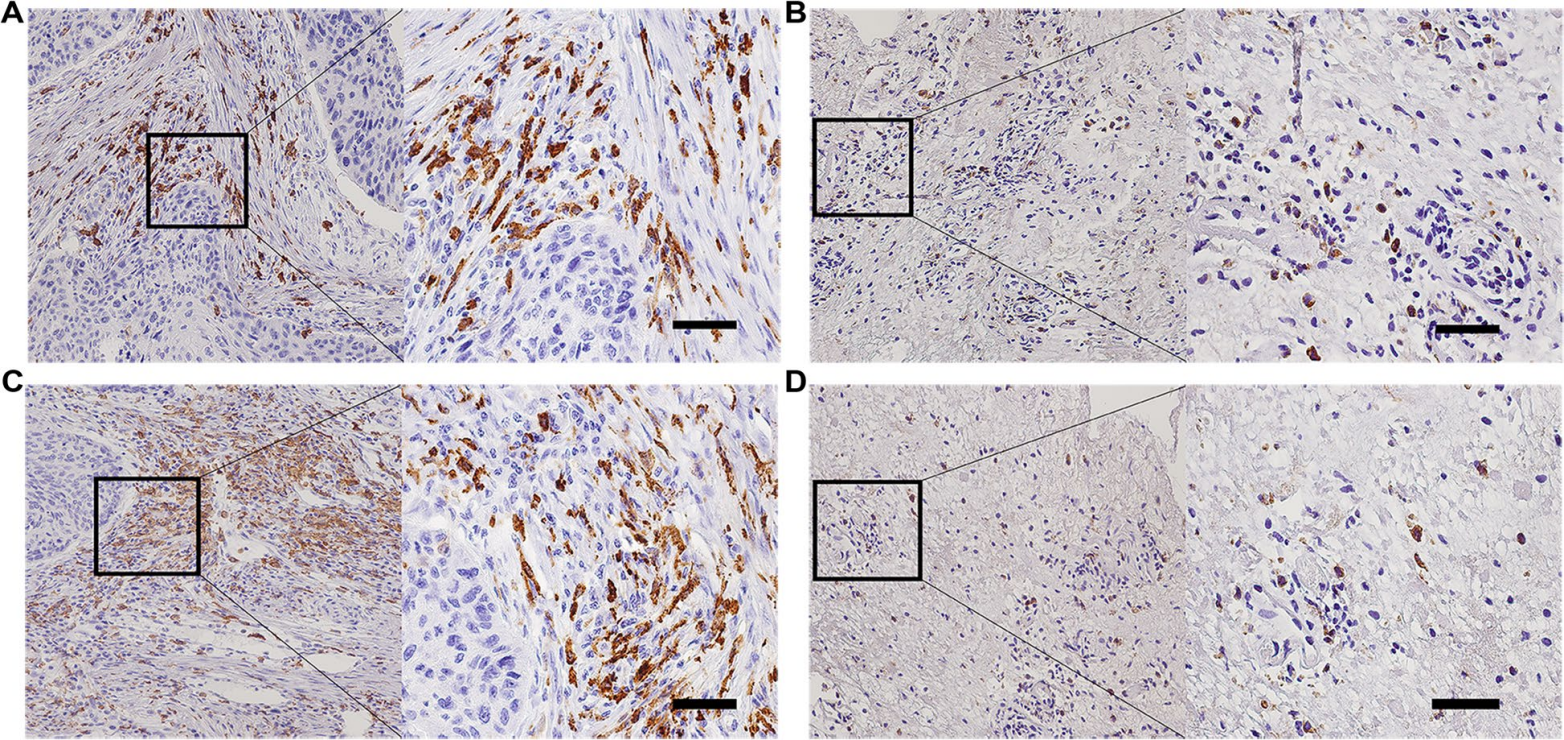
M2macrophage infiltrate the tissue of LSCC. Expressions of CD163 and CD206 were examined using Immunohistochemistry (IHC) inside LSCC carcinoma tissues. Scale bars: 50 μm (low magnification); 25 μm (high magnification). **A** Expression of CD163 in LSCC carcinoma tissues. **B** CD163 expression in adjacent tissues. **C** Expression of CD206 in LSCC carcinoma tissues. **D** CD206 expression in adjacent tissues

M2 macrophage markers were CD163 and CD206. To verify the difference in the expressions achieved by M2 markers in LSCC tissues and adjacent tissues (as control), thhe present study tested the expressions of the M2 markers of 104 LSCC cases. The results showed that there were considerable brown-yellow granules stained in the interstitial tissue of LSCC, Indicating a strong positive expression of CD163 and CD206 (Fig. 1A and C), while a small amount of pale yellow granules stained around the adjacent tissue, the quantity, and intensity of which contrasted with the expression of LSCC(Fig. 1B and D). This result suggested that there was a considerable amount of M2 macrophage infiltration in LSCC tumors, and the expression of M2 macrophage infiltration was significantly higher than that of normal tissues adjacent to LSCC, suggesting that M2 macrophage infiltration is dominant around LSCC tissues. To further verify the specificity of CD163 and CD206 expressions in the tissues of LSCC, we used RT-PCR to extract the expressions of CD163 and CD206 mRNA from LSCC tissues and adjacent tissues, to compare their relationship with clinicopathological parameters. As suggested from the results, the expressions of CD163 and CD206 were significantly high in LSCC tissues, displaying a close association with TNM stage (*P*-value < 0.05 was considered statistically significant) and clinical stage (*P*-value < 0.05 was considered statistically significant), instead of with age, sex and pathological classification (*P*-value > 0.05 was not statistically significant) (Table 1). Thus, the expression of M2 macrophages in LSCC tissue can predict the malignancy and prognosis of LSCC.

**Table 1:** Relationship between CD163 and CD206 expression and clinicopathological parameters of 104 LSCC patients

Characteristic	N	CD163	P	CD206	P
Sex					
Men	76	0.0521 ± 0.0323	0.3078	0.0464 ± 0.0317	0.5827
Women	28	0.0623 ± 0.0482		0.0519 ± 0.0492	
Age(y)					
< 58	62	0.0532 ± 0.0346	0.5935	0.0489 ± 0.0346	0.7370
≥ 58	42	0.0573 ± 0.0412		0.0463 ± 0.0408	
Locations					
Glottis	56	0.0496 ± 0.0204	0.1961	0.0422 ± 0.0118	0.0757
Supraglottis	40	0.0547 ± 0.0162		0.0483 ± 0.0176	
Subglottis	8	0.0605 ± 0.0198		0.0531 ± 0.0306	
T classification					
Tis	16	0.0457 ± 0.0213	0.0223*	0.0404 ± 0.0188	0.0362*
T1/T2	46	0.0496 ± 0.0170		0.0427 ± 0.0134	
T3/T4	42	0.0579 ± 0.0159		0.0503 ± 0.0173	
Lymph nodes					
N0	84	0.0504 ± 0.0307	0.0233*	0.0435 ± 0.0294	0.0307*
N1-3	20	0.0605 ± 0.0123		0.0534 ± 0.0140	
Grading					
Well	42	0.0485 ± 0.0174	0.1913	0.0462 ± 0.0172	0.4450
Moderately	34	0.0553 ± 0.0171		0.0471 ± 0.0158	
Poorly	28	0.0546 ± 0.0188		0.0421 ± 0.0159	
Pathological stage					
0, I, II	62	0.0486 ± 0.0181	0.0065*	0.0421 ± 0.0149	0.0142*
III, IV	42	0.0579 ± 0.0159		0.0503 ± 0.0173	

Relationship between the expression of CD163 and CD206 and clinicopathological parameters of 104 LSCC patients. Pathological staging was according to The International American Joint Committee on Cancer (AJCC-8). The “*” symbol indicates a statistically significant difference

### Macrophages co-cultured with LSCC cells successfully induced M2 polarization of macrophages

**Fig. 2:**
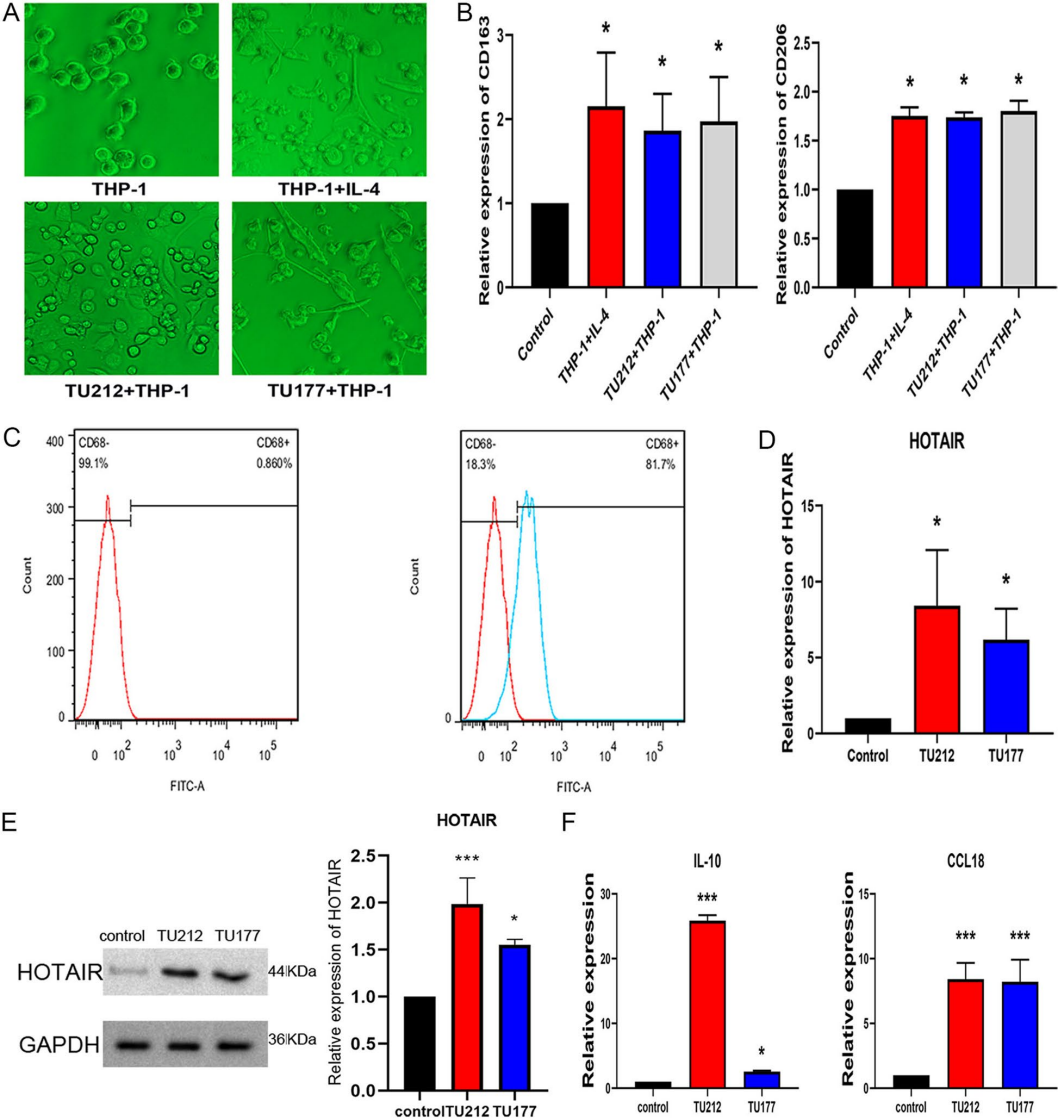
Macrophage cells displayed the M2 phenotype when the co-culture was achieved by using LSCC cells. Macrophage was co-cultured with TU212 cells or TU177 cells for 48 h. **A** Inverted microscopy images the morphology of macrophage. **B** Expression of CD163 and CD206 mRNA was detected by RT-PCR. **C** Characterization of macrophage phenotype by cell surface markers. Macrophage cells were stained in terms of CD68. Cells were gated in terms of CD68 representative survey results were presented. **D** and **E** The expression of lncRNA HOTAIR in macrophages was examined by RT-PCR and Western Blotting. **F** CCL18 and IL-10 levels in macrophage supernatants under the ELISA-based measurement. Data are expressed as mean ± SEM, *n* = 3 independent experiments. (*p < 0.05, *** *p* < 0.001 were considered statistically significant). Original gels are presented in Supplementary Fig. 2-E-HOTAIR and Fig. 2-E-GAPDH

To validate the phenotype of macrophage close to LSCC cells, THP-1 macrophage cells were co-cultured with TU212 cells or TU177 cells for 48 h. The morphology of macrophage was observed irregularly under an inverted microscope. The result showed that the size increased, the pseudopodium were extended and interlaced (Fig. 2A), consistent with the morphology of IL-4 induced M2 phenotype macrophage. Thus, THP-1 cells have been transformed into M2 macrophage in morphologically. The expression levels of CD163 and CD206 were significantly elevated in comparison to the control (Fig. 2B). The result of flow cytometry showed that the CD68 cell population grew noticeably when co-cultured with TU212 or TU177 cells (Fig. 2C). Moreover, in article, the expression level of lncRNA HOTAIR in macrophages significantly increased after being co-cultured with TU212 cells or TU177 cells (Fig. 2D and E). In the meantime, increased levels of CCL18 and IL-10 proteins were detected by ELISA (Fig. 2F). The above results indicated that macrophage around TU212 cells or TU177 cells largely showed the phenotype of M2. Furthermore, the expression of lncRNA HOTAIR significantly increased in macrophages.

### LncRNA HOTAIR secreted through exosomes improved M2 phenotype polarizing process

**Fig. 3:**
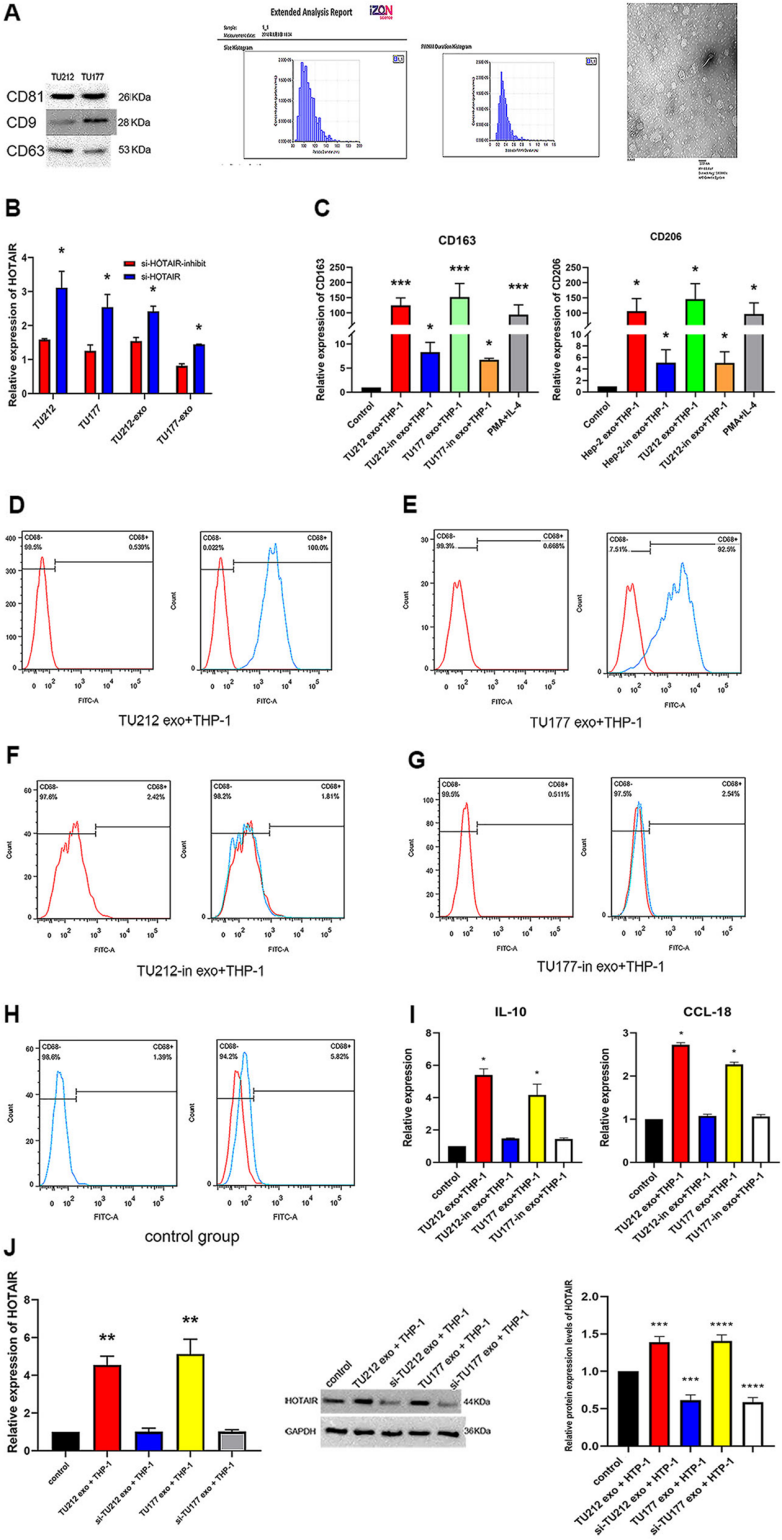
LncRNA HOTAIR secreted via exosomes promoted polarization of the M2 phenotype. Exosomes (TU212-Exo and TU177-Exo) were purified for NTA, Electron microscopic and WB analysis. WB analysis for exosomal proteins CD9, CD81 and CD63. NTA analysis of TU212-Exo and TU177-Exo isolated by ultracentrifugation, exosomes nanoparticles relative concentration; Electron microscopic image of TU212-Exo and TU177-Exo. Original gels are presented in Supplementary Fig. 3-A-CD9,Fig. 3-A-CD81 and Fig. 3-A-CD63. **B** LncRNA HOTAIR was analyzed by performing RT-PCR in TU212 cells or TU177 cells transfected with si-HOTAIR inhibitor, and exosomal lncRNA HOTAIR extracted from TU212-in or TU177-in were also detected. **C** CD163 and CD206 were detected by using RT-PCR in macrophage treated with TU212-Exo, TU177-Exo, TU212-in-Exo and TU177-in-exo. **D**, **E**, **F**, **G**, and **H** Characterization of phenotypes of macrophage by cell surface markers. M2 Macrophages were stained for CD68. Cells were gated for CD68. A representative analysis was presented in TU212-Exo and TU177-Exo, TU212-in-Exo, and TU177-in-Exo. RT-PCR and WB analysis for lncRNA HOTAIR in TU212-Exo and TU177-Exo, TU212-in-Exo, and TU177-in-Exo. Densitometry suggested relative protein expression normalized for GAPDH. Data are shown as mean ± SEM, *n* = 3 independent experiments. (*p < 0.05, **p < 0.01, *** *p* < 0.001 were considered statistically significant). Original gels are presented in Supplementary Fig. 3-K-HOTAIR and Fig. 3-K-GAPDH. **I** CCL18 and IL-10 levers in supernatants of macrophage, measured by ELISA. **J** and **K** RT-PCR and WB analysis for lncRNA HOTAIR in TU212-Exo and TU177-Exo, TU212-in-Exo, and TU177-in-Exo

To investigate the relationship of exosomal lncRNA HOTAIR and M2 macrophage, si-HOTAIR inhibitor was transfected into TU212 cells or TU177 cells by liposomes. Exosomes were isolated from the conditioned media after 48 h. As depicted in Fig. 3A, electron microscopy revealed typical rounded particles ranging from 30 to 150 nm in diameter, and NTA exhibited a similar size distribution of exosomes. Western blotting assay of proteins extracted from exosomes confirmed the presence of the exosomal proteins CD9, CD81, and CD63 (Fig. 3A). Then, the expression of exosomal lncRNA HOTAIR extracted from TU212 cells or TU177 cells after transfection was detected. Thus, it is suggested that the expression of exosomal lncRNA HOTAIR significantly increased in accordance with the expression of TU212 cells or TU177 cells, and it was down-regulated in the transfection with si-HOTAIR inhibitor (*p < 0.05 compared with the control treatments) (Fig. 3B). The above results suggested that exosomes with high or low expression lncRNA HOTAIR were successfully constructed.

To verify the lncRNA HOTAIR effect on macrophage polarization via exosomes, macrophage was incubated with pre-constructed low/high expression lncRNA HOTAIR exosomes for 48 h. CD163 and CD206 mRNA expression levels were up-regulated. The opposite was the low expression lncRNA HOTAIR group, in which the down-regulated expressions of CD163 and CD206 were observed (Fig. 3C). Consistent with the mentioned observation, CD68 cells markedly increased in number (Fig. 3D and E), compared with the low expression lncRNA HOTAIR group (Fig. 3F, G, and H). Besides, by ELISA, increased levels of CCL18 and IL-10 in supernatant were detected (Fig. 3I). Moreover, the expression level of lncRNA HOTAIR was elevated significantly in the macrophage cells incubated with high expression lncRNA HOTAIR exosomes, as opposed to the low expression HOTAIR exosomes (Fig. 3J and K). Thus, lncRNA HOTAIR was carried by exosomes from TU212 cells or TU177 cells to macrophage cells, which led to the promotion of M2 polarization.

### Exosome lncRNA HOTAIR polarized M2 macrophages by down-regulating PTEN expression and activating PI3K signaling pathway

**Fig. 4:**
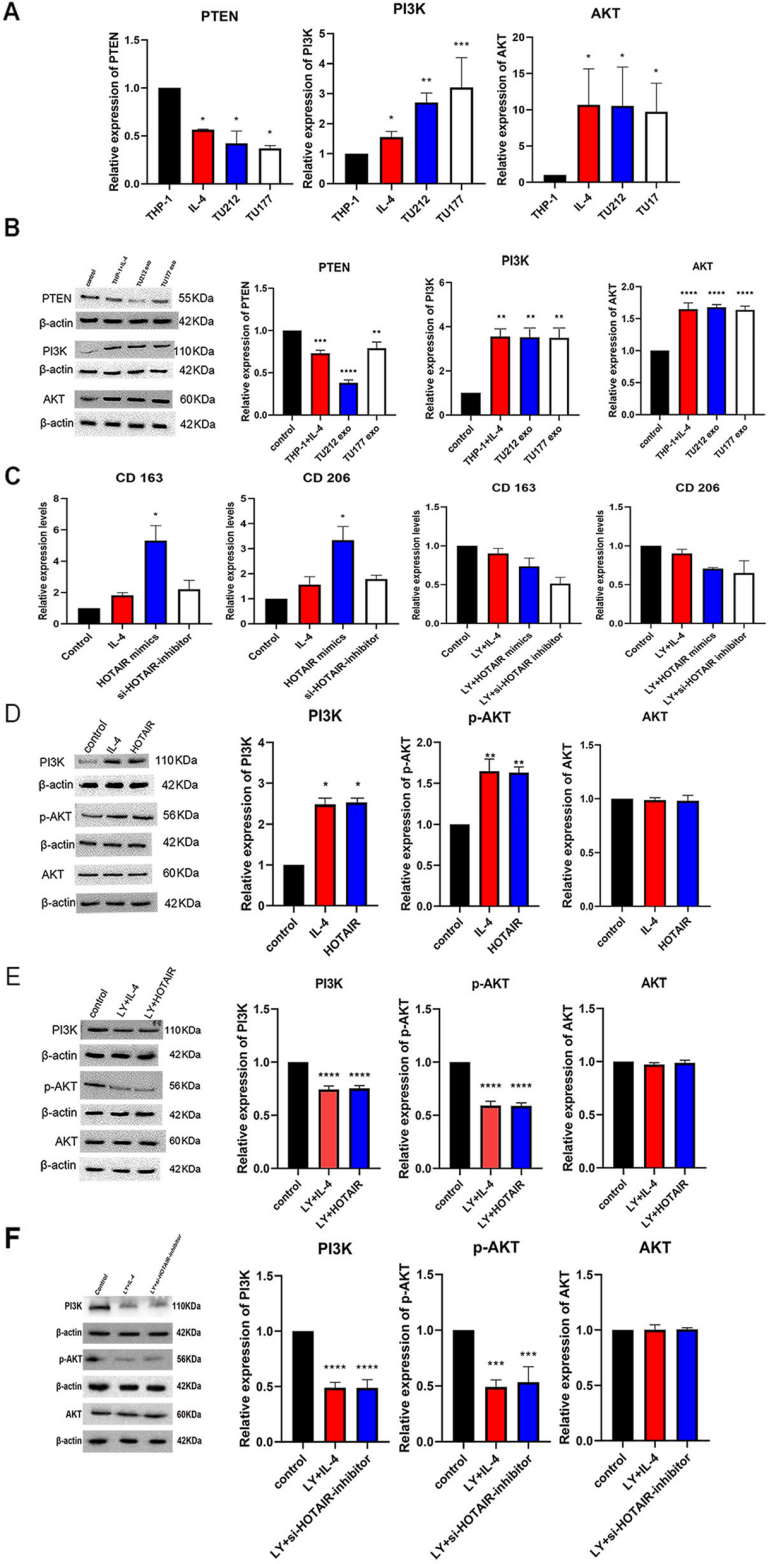
Exosomal lncRNA HOTAIR polarized the M2macrophage via down-regulating PTEN expression and activating a PI3K signaling pathway. Macrophages were incubated with exosomes extracted from TU212 cells or TU177 cells for 48 h, PTEN, PI3K and AKT in macrophage were tested by performing RT-PCR. **B** PTEN, PI3K and AKT protein in macrophage incubated with exosomes of TU212 cells and TU177 cells were performed by using WB. Original gels are presented in Supplementary Fig. 4-B-AKT, Fig. 4-B-PI3K, Fig. 4-B-PTEN, Fig. 4-B-β-actin (AKT), Fig. 4-B-β-actin (PI3K) and Fig. 4-B-β-actin (PTEN). **C** RT-PCR for CD206 and CD163 mRNA were measured in macrophage transfected with lncRNA HOTAIR mimics, post-transfected with PI3K inhibitor LY294002 and si-lncRNA HOTAIR inhibitor. Macrophages were transfected with lncRNA HOTAIR mimics, PTEN, p-AKT, and AKT protein in macrophage were detected by WB. **D** and **E** Macrophages were transfected with lncRNA HOTAIR mimics and post-treated with PI3K inhibitor LY294002, the differential expressions of PTEN, p-AKT, and AKT protein were quantified by performing WB. Original gels are presented in Supplementary Fig. 4-D-AKT, Fig. 4-D-PI3K, Fig. 4-D-p-AKT, Fig. 4-D-β-actin (AKT), Fig. 4-D-β-actin (PI3K) and Fig. 4-D-β-actin(p-AKT). Original gels are presented in Supplementary Fig. 4-E-AKT, Fig. 4-E-PI3K, Fig. 4-E-p-AKT, Fig. 4-E-β-actin (AKT), Fig. 4-E-β-actin (PI3K) and Fig. 4-E-β-actin (p-AKT). **F** Macrophages were transfected with si-lncRNA HOTAIR inhibitor, the differential expressions of PTEN, p-AKT, and AKT protein were detected by performing WB. Original gels are presented in Supplementary Fig. 4-F-AKT, Fig. 4-F-PI3K, Fig. 4-F-p-AKT, Fig. 4-F-β-actin (AKT), Fig. 4-F-β-actin(PI3K) and Fig. 4-F-β-actin (p-AKT)

To confirm that tumor-derived exosomes activate the PI3K pathway to promote polarization of macrophage via lncRNA HOTAIR, macrophage was treated with exosomes extracted from TU212 cells or TU177 cells for 48 h, and the expressions of PTEN, PI3K, and AKT in macrophage were detected. As depicted in Fig. 4A and B, incubation with exosomes down-regulated the expression of PTEN and up-regulated the expressions of PI3K and AKT, which displayed a negative relationship with the up-regulation of HOTAIR expression (Fig. 4A and B).

To further validate the mechanisms of exosomal lncRNA HOTAIR in the induction of macrophage polarization, lncRNA HOTAIR mimics and si-lncRNA HOTAIR inhibitor were transfected into macrophage to examine the activation of lncRNA HOTAIR/PI3K pathway. The expressions of CD206 and CD163 mRNA were down-regulated in macrophage transfected with si-lncRNA HOTAIR inhibitor and PI3K inhibitor LY294002, as opposed to those transfected with lncRNA HOTAIR mimics (Fig. 4C). As depicted in Fig. 4D, overexpression of lncRNA HOTAIR in macrophage significantly promoted the expressions of PI3K, phosphorylation of AKT, and AKT protein. Moreover, The expression of PI3K, phosphorylation of AKT and AKT protein in macrophage transfected with lncRNA HOTAIR mimics post-treated with PI3K inhibitor LY294002 was further examined. As depicted in Fig. 4E, the expressions of PI3K, phosphorylation of AKT protein were down-regulated in PI3K knocked down groups. The results were consistent with macrophage transfected with lncRNA HOTAIR inhibitor (Fig. 4F). On the whole, exosomal lncRNA HOTAIR activates PI3K/p-AKT/AKT signaling pathway to induce macrophage M2 polarization by down-regulating PTEN expression in macrophage.

Densitometry indicates relative protein expression normalized for β-actin. Data are expressed as mean ± SD of three independent experiments (*p < 0.05, **p < 0.01, *** *p* < 0.001, ****p < 0.0001 were considered statistically significant).

### M2 macrophage induced by exosomes promote the proliferation, migration, and EMT of LSCC cells

**Fig. 5:**
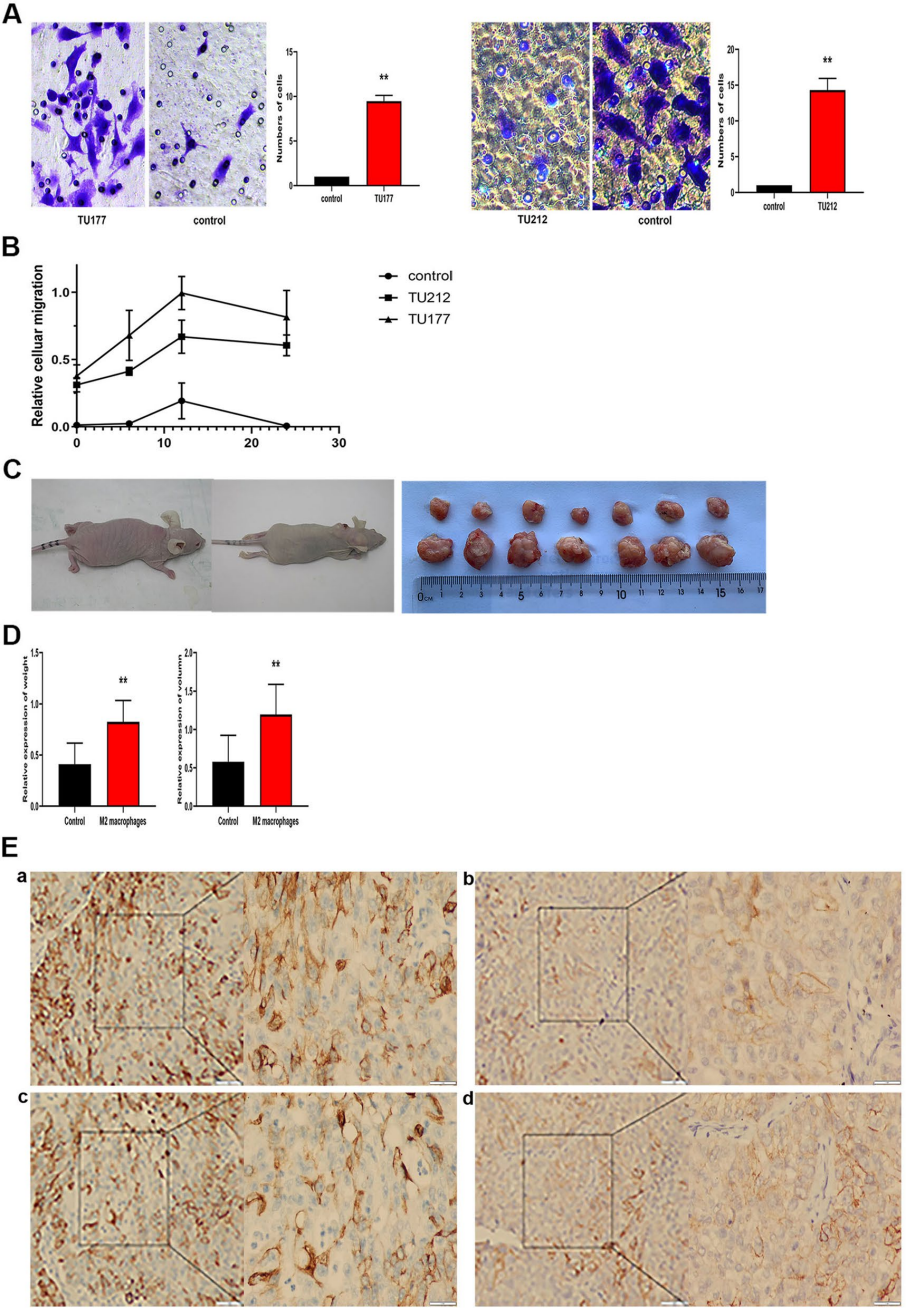
M2 macrophage induced by exosomes promote the proliferation, migration, and EMT of LSCC cells. TU212 cells and TU177 cells were co-cultured with macrophage under the treatment tumor-derived exosomes, **A** Migration capacity of LSCC cells(TU212 cells and TU177 cells) co-cultured with macrophage treated with exosomes was examined using the in vitro Transwell co-culture system. Representative photographs of migratory cells on the membrane coated with Matrigel (× 100 magnification) are generated. **B** Cell proliferation assay of LSCC cells treated with the culture medium of exosomes-treated M2 macrophage for 6 h, 12 h and 24 h. **C** Representative images of LSCC cell-xenografted in nude mice that resulted from co-injected with exo-treated macrophage cells or non-treated macrophage cells. **D** Volumes and weights of nude mice were compared after the injection of exosomes-treated or non-treated macrophages into the tumor. **E** The effect of the supernatants of macrophage transfected with exosomes on the EMT of LSCC cells was investigated by performing IHC. The data are expressed as mean ± SD of three independent experiments (*p < 0.01, **p < 0.05 was considered statistically significant)

To investigate the functions of exo-treated macrophage in the tumor micro-environment, TU212 cells and TU177 cells were co-cultured with M2 macrophage induced by tumor-derived exosomes. As indicated from the results of Migration Transwell assays, TU212 cells and TU177 cells significantly increased the migration in comparison with the control (Fig. 5A). Besides, The culture medium of exo-treated M2 macrophage was added to TU212 cells and TU177 cells, and LSCC cells were also more significantly proliferated (Fig. 5B).

In vivo, a comparison was conducted on the tumor volumes and weight of LSCC cell-xenografted mice co-injected with non-treated macrophage cells or exo-treated macrophage cells. Through the co-injection with exo-treated macrophage cells, significantly larger tumors were caused (Fig. 5C and D). Moreover, the expression levels of epithelial cell marker (E-cadherin) were down-regulated, and the expression levels of mesenchymal cell markers (N-cadherin) were up-regulated (Fig. 5E). As revealed from the mentioned results, macrophage polarized via LSCC cell-derived exosomes facilitated LSCC proliferation, migration and EMT.

## Discussion

Micro-environment critically impacts tumor development and metastasis, and the tumor cells contribute to their development by changing the surrounding environment in numerous manners [17]. The macrophage can be transformed into the M2 phenotype by the action of tumor cells and promote tumor proliferation [18]. In hepatocellular carcinoma, M2 macrophage is capable of promoting HCC malignant transformation and progression more significantly than M1 macrophage by up-regulating the expressions of vascular endothelial growth factor A, MMP-9 in the tumor micro-environment, which can be considered a vital factor for poor prognosis in HCC cases [19]. In hemangioma and squamous cell carcinoma [20] and non-small cell lung carcinoma [21], the majority of macrophages distributed in the tumor micro-environment comprise M2 polarization, and the macrophage is polarized to M2 type, which to some extent adversely affects tumor progression and prognosis. Accordingly, M1 macrophage inhibits tumor immunity, whereas M2 macrophage is closely related to tumor invasion and metastasis. In this experiment, considerable M2 macrophage infiltration was detected in the interstitial of LSCC tissue, not consistent with the expression of M2 macrophage in the normal tissue, demonstrating that M2 macrophage infiltration was dominant around the tissue of LSCC. Furthermore, the experimental results suggested that M2 macrophage expression in LSCC tissues is closely related to the TNM stage and clinical stage, whereas it is not related to age, sex and pathological classification. Thus, it is further confirmed that the expression of M2 macrophage can predict the malignancy and prognosis of LSCC.

Tumor cells enhance malignant tumor progression by releasing exosomes, which act in a paracrine or endocrine manner close or distant to mononuclear macrophage, promote macrophage to M2 polarization, and produce high levels of inflammatory factors that can facilitate tumor progression and EMT. In this experiment, LSCC cells were co-cultured with macrophage, and the result suggested that it can polarize macrophage to M2 polarization. By contributing high or low expression lncRNA HOTAIR of LSCC cells, the secreted lncRNA HOTAIR were verified to be successfully transferred into macrophage by exosomes and can induce macrophage to M2 polarization. Moreover, polarized M2 macrophage can promote LSCC cells’ proliferation, migration and EMT in vitro and vivo. The above results suggested that exosomal lncRNA HOTAIR are indispensable to the tumor micro-environment and act as a vital messenger that mediated the crosstalk between cells.

The signaling pathway of phosphoinositide 3-kinase (PI3K) is a pathway altered in high frequency. It is partially involved when myeloid cells are infiltrating in tumors. Besides, it is capable of regulating tumor growing and developing processes based on the promotion of neutrophils polarizing process, the control of immune suppressing process, as well as the mediation of integrin activating process. In SHIP-deficient macrophage, IL-4-induced activation of M2 macrophage requires crosstalk between PI3K activating process and STAT6 [22, 23]. Activation of M2 polarization requires AKT activating process since AKT inhibiting process abolished M2 gene up-regulation [24–26]. Although PI3K subunits exhibited obvious functions, a certain redundancy in these subunits was presented in a number of macrophage activating process fields [27, 28]. Moreover, tensin homolog (PTEN), phosphatase and lipid phosphatase under the regulation of PI3K were involved in macrophage polarizing process, suggesting the PI3K/AKT signaling pathway as the center node regulating macrophage polarizing process [29, 30]. Through the conversion of PI (3,4,5) P3 into PI (4,5) p2, PTEN antagonizes PI3K, both of which recruits AKT into the membrane of cell [31]. The PTEN loss leads to AKT activity increase while inhibiting the LPS response of macrophage [32]. PI (4,5) P2 generating process loses SHIP and increases the LPS response [33], converting PI(3,4,5) P3 to PI (4,5) P2, thus causing M2 skew in macrophage [34]. According to a previous study, HOTAIR under large expressing state can inhibit PTEN expression through PTEN methylation in LSCC. As revealed from the results here, LSCC-derived exosomal lncRNA HOTAIR can target the PTEN gene’s 3′ UTR, as well as suppress the relevant expressing process. Subsequently, the increased HOTAIR expressing state improved PI3K, p-AKT, and AKT expressions. For the mentioned reason, lncRNA HOTAIR is critical to facilitate M2 macrophage polarizing process based on the regulation of the PI3K/p-AKT/AKT pathway. For the mentioned reason, LSCC cells are capable of inducing macrophage to be polarized to M2 phenotypes through LSCC-derived exosomal lncRNA HOTAIR.

To sum up, LSCC-derived exosomal lncRNA HOTAIR was confirmed to induce the polarization of macrophage M2 by activating the PI3K/p-AKT/AKT pathway, thus facilitating LSCC cell to be migrated and invaded and to receive EMT. The mentioned information elucidates exosomal lncRNA HOTAIR system in LSCC progressing and stress such a pathway to be one diagnosis and therapy target for treating LSCC.

## Materials and methods

### Case samples and ethical statement

Collection of LSCC tissues: 104 samples of LSCC tissues and adjacent tissues (as the control) of cases in Otorhinolaryngology head and neck surgery in the Second Affiliated Hospital of Harbin Medical University during 2020 ~ 2021 were collected. All of the mentioned cases were diagnosed with LSCC by pathological preoperative, and these underwent the complete relevant surgical treatment. Pathological staging was according to The International American Joint Committee on Cancer (AJCC-8).

### Immunohistochemistry

LSCC tissues, adjacent tissues and LSCC cell-xenografted tumor were fixed, the paraffin sections were stained at 60 °C for 1 h before dewaxing, hydrogen peroxide closed endogenous peroxidase, antigen thermal repair, as well as normal serum closed. The primary anti-CD163 and anti-CD206, anti-N-cadherin and anti-E-cadherin 5 µg/ml (Abcam, USA) were added at 37 °C for 2 h(also able to be incubated overnight in a refrigerator at 4 °C). Subsequently, the biotinylated second antibody was added at 37 °C for 40 min. Triantibody(SAB complex) was added at 37 °C for 40 min. It was colored with DAB and observed under a microscope. Then, the process was timely terminated. Hematoxylin restrained at ambient temperature. Gradient alcohol dehydration again sealed the sheet, which was placed in a 60 °C oven to dry. The results were observed under a light microscope.

### Cell culture and treatment

Human laryngeal squamous cell lines TU212, TU177, and human mononuclear cell line THP-1 were purchased from the Chinese Academy of Sciences Cell Bank of Type Culture Collection. The cell lines were maintained at 37 °C in a humidified atmosphere of 5% CO2 with RPMI-1640 medium supplemented with 10% fetal calf serum (FBS) with 100 U/ml penicillin G and 100 μg/ml streptomycin sulfate.

To induce differentiation into macrophages, THP-1 cells (1 × 106/ml) were incubated for 24 ~ 48 h with 100 ng/ml PMA (Sigma, MO, USA), and PBS was rinsed three times to produce M0 macrophage. Before exosomes extraction, TU212 cells and TU177 cells were cultured for 48 h with serum without 10% FBS.

THP-1 cells (1 × 106) were planted in the lower chamber with 0.4 mm pores (catalog number140660; Thermo Fisher Scientific, Waltham, MA) in accordance with the Transwell chamber principle. After PMA was added for 24 h ~ 48 h, the cells adhered to the wall, TU212 cells and TU177cells (1 × 105) cells were planted in the insert chamber.

### Extraction and identification of exosomes

The cell lines were cultured in the normal medium until 80–90% confluent. Subsequently, the medium was replaced with RPMI 1640 with 10% exosome-depleted FBS. Next, cell culture medium was harvested after 3 days (30 ml) and then centrifuged at 3000 × g for 10 min to collect the supernatant. The supernatant was filtered with a 0.22 μm filter twice and then ultracentrifuged at 100,000 × g for 70 min (HITACHI, Japan) to collect the pellet. Afterwards, the pellet was suspended in 10 ml PBS and then ultracentrifuged at 100,000 × g for 70 min again. The collected exosomes were resuspended in 100ul PBS. The exosomes suspension was packed and preserved at -80 °C, and repeated freeze–thaw was avoided. All ultracentrifugation should be performed at 4 °C. When the supernatant was being absorbed rigorously, inhaling cells or cell fragments should be avoided.

Particle tracking and video capture software in the Nanosight LM10 system(Nanosight Ltd, Navato) were adopted to calculate the size and concentration of nanoparticles by measuring their Brownian motion speed, as an attempt to determine whether the extract was exosomes. Besides, the morphology of exosomes was observed under a transmission electron microscopy. (The exosome suspension was fixed with glutaraldehyde and then stained with 2% uranium).

### Transfection

Specific to the overexpression of lncRNA HOTAIR, TU212 cells, TU177 cells and macrophage cells were transfected with either lncRNA HOTAIR inhibitor plasmid(Source Bioscience) or HOTAIR mimics with Lipofectamine 2000 (Invitrogen, Carlsbad, CA). Next, the transfected cells were harvested and then collected after 48 h, and the lncRNA HOTAIR expression was analyzed.

### RT-PCR

In terms of LSCC tissue and adjacent tissue samples, cell samples and exosomes samples, total RNA was isolated with the miRNeasy Mini Kit (Qiagen) by complying with the producer’s instructions, and DNA was eliminated (Rnase-Free Dnase Set Qiagen). Reverse transcription was performed with the Omniscript RT Kit (Qiagen), and PCR was conducted with SYBR Green PCR Master Mix (Applied Biosystems, Foster City, CA). Expression data were uniformly normalized to the internal control U6, and the relative expression levels were examined using the 2-ΔΔCT method. The primers originated from Invitrogen (Carlsbad, CA).

The primer sequences used were as follows: CD206, CD163, PTEN, PI3K, and AKT.

**Table:** 

CD206 primer,	forward: 5’-GGGAAAGGTTACCCTGGTGG-3’
	reverse: 5’-GTCAAGGAAGGGTCGGATCG-3’
CD163 primer,	forward: 5’-GATATGGCTCAATGAAGTGAAGTG-3’
	reverse: 5’-AATAAAGGATGACTGACGGGATG-3’
PTEN primer,	forward: 5’-TGGAGGCTATCAACAAAGAATGG-3’
	reverse: 5’-TACA CAGG AGAT GGAGAAGTCG-3’
PI3K primer,	forward: 5’-GTTGGTGGCTGTTCTTACTGTC-3’
	reverse: 5’-CAAGTCTGGCTGGAATGATGC-3’
AKT primer,	forward: 5’-GTCATCGAACGCACCTTCCAT-3’
	reverse:5’-AGCTTCAGGTACTCAAACTCGT-3’
HOTAIR primer,	forward: 5’-CAAGTCTGGCTGGAATGATGC-3’
	reverse:5’-ATGGAGATGATAAGAAGAGCAAGG-3’
RNU6B primer,	forward: 5’-CGCAAGGATGACACGCAAATTC-3’
	reverse: 5’-AAAAATATGGAACGCTTCAC-3’

### Western blot

By using whole protease and phosphatase inhibitor cocktails (Sigma, USA), cells or exosomes were harvested process and the lysed in RIPA lysis buffer. After the 30 min centrifugating process at 15,000 × g, supernatant proteins concentrations were studied with the Bradford reagent (Bio-Rad, Hercules, CA). On the whole, 30 μg of protein for each specimen was electrophoresed inside SDS-PAGE gels and then transferred to one 0.22 μm PVDF membrane (Millipore, MA, USA). After blocking, the membranes were incubated using major antibodies at 4 °C throughout the night and then the incubating process by using the HRP-conjugated secondary antibody (1:5000, LI-COR Biosciences, USA) under ambient temperature. Lastly, the membranes were visualized using Thermo Pierce chemiluminescent (ECL). The primary antibodies employed experimentally included anti-CD9 (1:500; Santacruz, USA); anti-PTEN (1:1000; Abcam, USA); anti-p-AKT, anti-AKT, anti-PI3K (1:1000; Cell Signaling Technology, USA), and anti-GAPDH (1:10,000; Abcam, USA).

### Flow cytometry

For measuring the expression status of cell surface markers, cells were harvested, the suspended inside staining buffer (PBS supplemented by 1% FBS and 0.1% NaN3), and incubated for 30 min using FITC-conjugated anti-human CD68 (BD Biosciences, USA) at 4 °C. Then the Analyses of FACS Calibri flow cytometer (BD Biosciences) were conducted on the cells from the Centre for Cytometry and Fluorescence Microscopy Complutense University of Madrid (Spain).

### Embedded cell co-culture method

To perform cell migrating tests, this study employed 24-well plates and 8 μm Transwell inserts (Corning Life Science, MA, USA). First, THP-1 cells in 800 μl were inoculated in the lower chamber by using PMA 100 ng/ml for 24 ~ 48 h and TU212 cells or TU177 cells (105/ml) suspended in 200 μl serum-free medium were seeded in the upper chamber. After being cultured for 24 h-48 h, cells were then stained using 0.1% crystal violet and then counted. This study determined the migrated cell number with Six visual fields selected in a random manner.

### CCK8

For determining the effect exerted by exosomes induced-macrophage, this study used one cell counting kit-8(CCK8, Beyotime Biotechnology) by complying with the instructions of the producer. In brief, TU212 cells and TU177 cells were seeded within one 96-well plate(5 × 103cells/well), followed by the treatment by using the culture medium of exosomes induced-macrophage for another 48 h. Then, this study introduced 10 μl of CCK8 solution into the respective well, and by employing one microplate reader(Synergy HT, Biotech), the 450 nm absorbance was examined at 4 h, 6 h, and 8 h, respectively. The OD values were examined to indicate the population of the viable cell of the respective well.

### ELISA

The concentration of CCL18 and IL-10 inside supernatants of culture was examined using one Quantikine ELISA Tool (R&D Systems) following the instruction of the producer.

### Animal experiments

Male BALB/c nude mice (aged 4 weeks) (Institute of Zoology, Chinese Academy of Sciences, Beijing, China) were housed under a particular pathogen-free condition inside the Second Affiliated Hospital’s Animal Laboratory Unit, Harbin Medical University. The overall animal experimental processes gained approval from the Ethics Committee of Harbin Medical University. Besides, the experimental processes were conducted based on Institution’s instructions and animal experiment standards. A total of 14 male mice were randomly assigned into two groups (seven mice per group). TU212 cells (1 × 106 cells in 100 μl PBS) under the mixture with the conditioned macrophage stimulated by TU212-exo-treated or non-treated were injected into the nude mice back. Six weeks later, the tumor size was monitored by measuring width (W) and the length (L), and the volume(V) was estimated by: V = (L × W2) × 0.5.

### Statistical analysis

The statistics of this study were analyzed using GraphPad Prism 7.0 software (GraphPad Software, USA), followed by the display to be the mean and SEM. Statistics-related significant characteristic of groups was examined through one-way ANOVA test and two-tailed Student’s t-test. A *P*-value < 0.05 was considered statistically significant. The difference achieved statistical significance when the P-value was less than 0.05 and was represented by *P < 0.05, **P < 0.01, ***P < 0.001, and ****P < 0.0001. 

## Supplementary Information

**Additional file 1.** 

## Data Availability

The datasets used and/or analysed during the current study available from the corresponding author on reasonable request.
