# Supplementary material for: Exosomal lncRNA HOTAIR induce macrophages to M2 polarization via PI3K/ p-AKT /AKT pathway and promote EMT and metastasis in laryngeal squamous cell carcinoma
Source: BMC Cancer. 2022 Nov 24;22:1208. doi: 10.1186/s12885-022-10210-5 (PMC9686105; doi:10.1186/s12885-022-10210-5)
Supplement: Supplementary file 1 — Additional file 1. [file 12885_2022_10210_MOESM1_ESM.doc]

1. Originals of fig 2E

HOTAIR

GAPDH

2. Originals of fig 3A

CD 81

CD9

CD63

3. Originals of fig 3J

HOTAIR

GAPDH

4. Originals of fig 4B

PTEN

β-actin

PI3K

β-actin

AKT

β-actin

5. Originals of fig 4D

PI3K

β-actin

P-AKT

β-actin

AKT

β-actin

6. Originals of fig 4E

PI3K

β-actin

p-AKT

β-actin

AKT

β-actin

7. Originals of fig 4F

PI3K

β-actin

p-AKT

β-actin

AKT

β-actin
